# A phase I study of foretinib plus erlotinib in patients with previously treated advanced non-small cell lung cancer: Canadian cancer trials group IND.196

**DOI:** 10.18632/oncotarget.18753

**Published:** 2017-06-28

**Authors:** Natasha B. Leighl, Ming-Sound Tsao, Geoffrey Liu, Dongsheng Tu, Cheryl Ho, Frances A. Shepherd, Nevin Murray, John R. Goffin, Garth Nicholas, Shingo Sakashita, Zhuo Chen, Lucia Kim, Jean Powers, Lesley Seymour, Glenwood Goss, Penelope A. Bradbury

**Affiliations:** ^1^ Canadian Cancer Trials Group (Formerly NCIC Clinical Trials Group), Kingston ON, Canada; ^2^ Princess Margaret Cancer Centre/University Health Network, Toronto ON, Canada; ^3^ Ontario Cancer Institute, Toronto ON, Canada; ^4^ British Columbia Cancer Agency, Vancouver BC, Canada; ^5^ Juravinski Cancer Centre, Hamilton ON, Canada; ^6^ Ottawa Hospital Cancer Centre, Ottawa ON, Canada

**Keywords:** foretinib, erlotinib, MET, AXL, non-small cell

## Abstract

**Purpose:**

MET and AXL mediate resistance to EGFR TKI in NSCLC. Foretinib, a MET/RON/AXL/TIE-2/VEGFR kinase inhibitor may overcome EGFR kinase resistance. This dose escalation study combined foretinib and erlotinib in advanced pretreated NSCLC patients.

**Experimental Design:**

The primary endpoint was to define the RP2D of foretinib plus erlotinib as continuous oral daily dosing. Secondary objectives included safety, pharmacokinetics, response and potential biomarkers of response including *EGFR, KRAS* genotype, MET, AXL expression, and circulating HGF levels. Erlotinib (E100-150 mg) was commenced on day 1 cycle 1; if well tolerated, foretinib (F30-45 mg) was added on day 15 cycle 1, using standard 3+3 dose escalation.

**Results:**

Of 31 patients enrolled in 3 dose levels, 6 were inevaluable for DLT and replaced. DLT occurred in 3/15 patients at DL2 (E150 mg, F30 mg): Gr3 pain, mucositis, fatigue and rash. Cycle 1 DLT was not seen at DL3 (E150 mg, F45 mg) but 27% experienced dose reduction/interruption. Adverse events in ≥20% included diarrhea, fatigue, anorexia, dry skin, rash and hypertension. No PK interaction was seen with the combination. RP2D was defined as erlotinib 150 mg daily x 14 days with foretinib 30 mg added on day 15 (continuous dosing in 28-day cycles). Responses were seen in 17.8% of response evaluable patients (5/28). In 18 samples, baseline MET expression uncontrolled for *EGFR* genotype appeared associated with response. AXL expression was associated with neither *EGFR* mutation nor response.

**Conclusion:**

Combining foretinib and erlotinib demonstrated response in unselected advanced NSCLC but also incremental toxicity. Future development will require molecular patient selection.

## INTRODUCTION

Foretinib is a multitargeted kinase inhibitor, targeting MET, RON, AXL, TIE-2, VEGF receptors and ROS-1, with documented activity in papillary renal carcinoma and hepatocellular carcinoma [1–3]. Preclinical studies demonstrate potent inhibition of MET, AXL, RON, VEGF and ROS1 with IC_50_ values ranging from 1.8 to 8 nM [1, 3, 4]. The agent has also been studied in gastric and head and neck cancers with less activity, likely related to a lack of dependence on MET- and VEGF receptor-mediated signaling in most of these tumors [5, 6].

In lung cancer, MET remains an important and challenging target. High levels of MET protein expression, seen in half of non-small cell lung cancer (NSCLC) cases, are associated with poor prognosis [7]. The role of *MET* mutations in lung cancer, seen in up to 7% of adenocarcinomas, remains complex [8]. Germline MET mutations have been identified in patients with squamous carcinoma, smoking history and East Asian origin [9]. Semaphorin or juxtamembrane mutations may not lead to MET activation, while splice site mutations seen in 4% of lung adenocarcinomas and 2% of squamous cases may lead to MET activation via exon 14 skipping in *MET* mRNA with response to MET inhibitors [10]. MET amplification (MET/CEP7 ratio ≥5) is a rare independent finding in lung cancer (<0.5%) but is also associated with MET inhibitor sensitivity [10]. High *MET* copy number has also been detected in 5% of *EGFR* mutant cases in the setting of acquired EGFR kinase resistance, and even primary resistance to EGFR kinase inhibitors may be mediated via upregulation of hepatocyte growth factor (HGF)-MET signaling [11–13]. In preclinical studies, foretinib significantly increases sensitivity in *EGFR* mutant lung cancer cells with upregulated HGF and increased MET copy number when added to erlotinib [14]. AXL, another target of foretinib, is involved in signal transduction of growth factors (GAS6), proliferation and regulation of epithelial-to-mesenchymal transition in metastasis. Activation of AXL kinase has been associated with acquired resistance to EGFR kinase inhibitors in *EGFR* mutant lung cancer, with evidence for epithelial-to-mesenchymal transition in preclinical models and restored EGFR kinase inhibitor sensitivity upon AXL inhibition [15, 16].

Thus, the combination of foretinib, a potent MET and AXL inhibitor, with erlotinib therapy in NSCLC appears a rational and promising way to exploit potential synergism between agents and to overcome primary resistance as well as delaying the development of resistance to EGFR kinase therapy. Although response rates were higher in patients with *EGFR* mutant tumours, the use of erlotinib in molecularly unselected NSCLC patients after failure of one or two lines of chemotherapy demonstrated modest survival and quality of life benefit compared to placebo in all patients [17, 18]. Given the major unmet need for better treatments in this population, the objectives of this dose escalation study were to define the recommended phase II dose (RP2D) of daily continuous oral dosing of foretinib plus erlotinib in advanced, pretreated NSCLC patients, to assess safety and tolerability, pharmacokinetic, preliminary antitumor activity and pharmacodynamic data with the combination.

## RESULTS

Over a three-year period (January 2010 to January 2013), 31 patients were accrued at 3 dose levels at the 4 participating centers (Table 1). The median age of the study population was 63, (range 36 to 74 years). Eleven (35%) were female, and 23% and 74% were ECOG PS 0 or 1. All had received prior chemotherapy, 18 prior radiation (58%) and 9 other therapy. The median number of prior chemotherapy regimens received was 2, with 10 (32%) entering the study after 1 line of therapy and 19 (61%) after 2 lines. Two were found post-registration to have received 3 lines of therapy and have been included in this analysis. The majority (93%) had adenocarcinoma subtype, one had large cell carcinoma with neuroendocrine differentiation and another had squamous carcinoma.

**Table 1:** Adverse events >= grade 3 and/or occurring in >15% of patients and at least possibly related to foretinib (N=31)

Adverse Event	Dose Level 1 (N=9)				Dose Level 2 (N=15)				Dose Level 3 (N=7)			
Grade	1	2	3	4	1	2	3	4	1	2	3	4
Non-laboratory Events												
Vision/eye disorders	2	1			1				1			
Abdominal pain/distention	1				1				2	4	1	
Diarrhea					3	4	2		5	2		
Mucositis	1				4		1					
Nausea					1	1	1					
Fatigue		2			2	5	2		1	1	1	
Anorexia					5	3			3			
Myalgia/arthralgia					2				1		1	
Neck pain							1					
Syncope											1	
Hoarseness									3			
Dry skin	1				6	1				1	1	
Nail changes	2				1							
Rash**	3	1			2	5	3		1	4	3	
Palmar-plantar erythrodysesthesia		1			2	1						
Paronychia						1			1		1	
Purpura											1	
Hair growth (slow)					2	1						
Hypertension		1	1		1	2	4		1	1	2	
Laboratory events												
Anemia	7				9	1			6			
Lymphopenia	2	3	1		7	3	3		3	4		
Thrombocytopenia	1	1			3				1			
Creatinine	2				5				2			
Hypoalbuminemia	5	1			9	3	1		3	4		
Alkaline phosphatase	2	1			7	1						
AST	6	1			12				6	1		
Hyperbilirubinemia					4	1			1			
Hypocalcemia							1					
Proteinuria					2		1					

**Multiple AE rash events (pupura, rash acneiform and rash maculo-papular) were combined. Rash events for DL3 sum to more than N as one patient could have multiple rash events.

Six patients were inevaluable for DLT and were replaced. Three of 6 did not start foretinib (disease progression 2, intercurrent illness 1), one developed disease progression after only 6 days of foretinib, another did not tolerate erlotinib, delaying foretinib start and another took half dose foretinib in error during cycle 1. This last patient did go on to receive 7 cycles of protocol therapy and was evaluable for response. In the first dose level, three of four patients entered were evaluable and no DLT was seen. In the second dose level, three of four accrued were evaluable, and one developed DLT, grade 3 rash requiring treatment delay and erlotinib dose reduction in cycle 2. An additional 4 patients were entered at the expanded second dose level, with no further DLT seen. At the third dose level, four patients were accrued with no DLT. But because of initial pharmacokinetic analysis suggested a possible two-fold increase in foretinib exposure over expected, an additional three patients were entered at the expanded third dose level to further characterize PK, none of whom experienced DLT. However confirmation of a potential pharmacokinetic interaction, and concern regarding intolerable toxicities beyond cycle 1 at the third dose level led to a further expansion of the second dose level, (30 mg foretinib plus 150 mg erlotinib). Three of four patients entered to the expanded dose level were inevaluable for DLT and were replaced. Of the remaining 4 evaluable patients, two had DLT in cycle 1, grade 3 diarrhea, mucositis, fatigue and neck pain resulting in hospitalization, treatment delay and dose reduction of both agents for one patient, and grade 3 fatigue and nausea resulting in a two week treatment delay for the second patient. A further 4 patients were entered into the expanded dose level 1 (30 mg foretinib + 100 mg erlotinib), one of whom progressed in CNS and was replaced. Another had grade 3 diarrhea in cycle 1 but this was deemed inadequately managed and did not meet the definition of DLT.

On data review by the trial committee, including toxicity in cycle 1 and subsequent cycles and pharmacokinetic data, the second dose level was determined to be the recommended phase II dose, foretinib 30 mg daily plus erlotinib 150 mg daily after a 2 week run in of single agent erlotinib.

### Toxicity

Adverse events deemed at least possibly related to either foretinib or erlotinib are summarized in Table 1 by dose level. The most common toxicities included rash, pruritus, dry skin, fatigue, diarrhea, hypertension, lymphopenia, thrombocytopenia and transaminitis, anorexia, nausea and abdominal pain. DLT are listed above.

### Response

Of the 31 patients enrolled, 28 were evaluable for response, and 3 did not have repeat assessment of their disease. Five experienced a partial response (17.8%), with a median duration of response of 10.8 months (range 3.6-17 months). Thirteen had stable disease, median duration 4.8 months (range 2.4-15.4 moths) and 10 had disease progression as their best response.

Although trial eligibility mandated submission of tissue for genotyping for all patients, only 11/31 patients had sufficient tissue for successful analysis, with 3 *EGFR* mutations and 4 *KRAS* mutations identified, (all mutually exclusive). Of 5 patients that experienced partial response to therapy, 2 had documented *EGFR* mutations, 1 had wild-type *EGFR* and 2 unknown *EGFR* genotype. Two of three patients with identified *EGFR* mutations experienced response. All 4 patients with *KRAS* mutations identified had stable disease as their best response to treatment.

### Dose intensity, treatment duration

Dose intensity of each agent and number of cycles received by dose level is shown in Table 2. As dose level increased, dose intensity and treatment duration decreased. Four of nine in the first dose level required no dose delay or reduction; 2 of 15 in the second dose level required no modifications of either erlotinib or foretinib and no patients in the third dose level received full doses of planned treatment without at least one missed dose or dose reduction of erlotinib, foretinib or both. Reasons for patients discontinuing study therapy included 20/31 stopping for disease progression, 2 for death, 3 for unrelated intercurrent illness, 5 (16%) for treatment-related adverse events and another for noncompliance with protocol therapy. The treatment-related adverse events included grade 3 proteinuria, grade 2 blurred vision, grade 2 rash and fatigue, grade 3 hypertension and rash (grade 2), and grade 3 myalgia and hypertension (grade 2).

**Table 2:** Treatment delivery (n=31)

Dose Level	DL1 (N=9)	DL2 (n=15)	DL3 (N=7)
Foretinib/Erlotinib (mg)	30/100	30/150	45/150
Median number of cycles delivered (range)	2 (1-14)	4 (1-22)	4 (1-8)
% receiving >=90% planned dose intensity Foretinib Cycle 1 Foretinib Cycle >= 2 Erlotinib	100% 50% 78%	67% 31% 40%	86% 0 43%
Patients with no dose modification of either drug (omitted, delayed)	4 (44%)	2 (13%)	0

### Pharmacokinetic studies

Pharmacokinetic data are shown in Table 3 and were available for 27 patients for erlotinib and 24 patients for foretinib. Initial data suggested a possible pharmacokinetic interaction between foretinib and erlotinib, with wide confidence intervals suggesting a two-fold increase in foretinib exposure over what was expected based on single agent studies. This led to expanded dose levels, but with additional patients studied, further analysis did not confirm any pharmacokinetic interaction between erlotinib and foretinib (see Table 3).

**Table 3:** Pharmacokinetic analysis by dose level for erlotinib (N=27) and foretinib (N=24)

Dose Level	Parameter	Erlotinib D14 (DL1 N=6; DL2 N=9; DL3 N=7)	Erlotinib D28 (DL1 N=7; DL2 N=11; DL3 N=6)	Foretinib D28 (DL1 N=7; DL2 N=11; DL3 N=6)
1	AUCt (ng*hr/mL)	30104 (SD 14925)	35928 (SD23409)	728 (SD 221)
	Cavg (ng/mL)	1254 (SD 622)	1497 (SD 975)	30.35 (SD 9.23)
	Clast (ng/mL)	1106 (SD 710)	1300 (SD 861.44)	27.29 (SD 10.66)
	Clearance (mL/hr)	1717 (SD 2219)	2035 (SD 3328)	25153 (SD 32679)
	Cmax (ng/mL)	1719 (SD 624)	2387 (SD 1707)	43.37 (SD 17.6)
	Cmax Dose Norm (ng/mL/mg)	17.2 (SD 6.24)	23.87 (SD 17.07)	1.45 (SD 0.59)
	Cmin (ng/mL)	815 (SD 641)	1092 (SD 894)	22.79 (SD 7.74)
	Tmax (hr)	7.67 (SD 8.5)	2.29 (SD 1.7)	3.14 (SD 2.73)
	Tmin (hr)	4.17 (SD 9.71)	11.2 (SD 12.07)	12.29 (SD 11.34)
2	AUCt (ng*hr/mL)	35190 (SD 12164)	36221 (SD 16365)	681 (SD 337)
	Cavg (ng/mL)	1466 (SD 507)	1579 (SD 643)	29.17 (SD 13.58)
	Clast (ng/mL)	1137 (SD 419)	1343 (SD 687)	24.12 (SD 9.34)
	Clearance (mL/hr)	5124 (SD 6536)	4567 (SD 7325)	29543 (SD 30935)
	Cmax (ng/mL)	2335 (SD 770)	2645 (SD 974)	44.79 (SD 22.34)
	Cmax Dose Norm (ng/mL/mg)	15.6 (SD 5.13)	17.64 (SD 6.49)	1.49 (SD 0.74)
	Cmin (ng/mL)	1047 (SD 437)	1293 (SD 688)	22.47 (SD 10.61)
	Tmax (hr)	2.22 (SD 1.48)	2.64 (SD 2.25)	3.55 (SD 2.38)
	Tmin (hr)	13.33 (SD 12.65)	13.27 (SD 12.34)	13.36 (SD 12.23)
3	AUCt (ng*hr/mL)	35545 (SD 16948)	40765 (SD 16673)	1010 (SD 415)
	Cavg (ng/mL)	1548 (SD 621)	1699 (SD 695)	42.07 (SD 17.31)
	Clast (ng/mL)	1247 (SD 565)	1283 (SD 697)	34.22 (SD 17.3)
	Clearance (mL/hr)	4792 (SD 2292)	4571 (SD 2760)	49768 (SD 16023)
	Cmax (ng/mL)	2237 (SD 620)	2472 (SD 717)	55.85 (SD 17.24)
	Cmax Dose Norm (ng/mL/mg)	14.91 (SD 4.14)	16.48 (SD 4.78)	1.21 (SD 0.4)
	Cmin (ng/mL)	1027 (SD 507)	1244 (SD 725)	31.96 (SD 18.16)
	Tmax (hr)	3.86 (SD 2.19)	2.33 (SD 1.86)	3.5 (SD 3.02)
	Tmin (hr)	4.71 (SD 8.77)	16.17 (SD 12.14)	12.0 (SD 13.15)

DL: dose level; SD: standard deviation

### Pharmacodynamic studies

**Figure 1:**
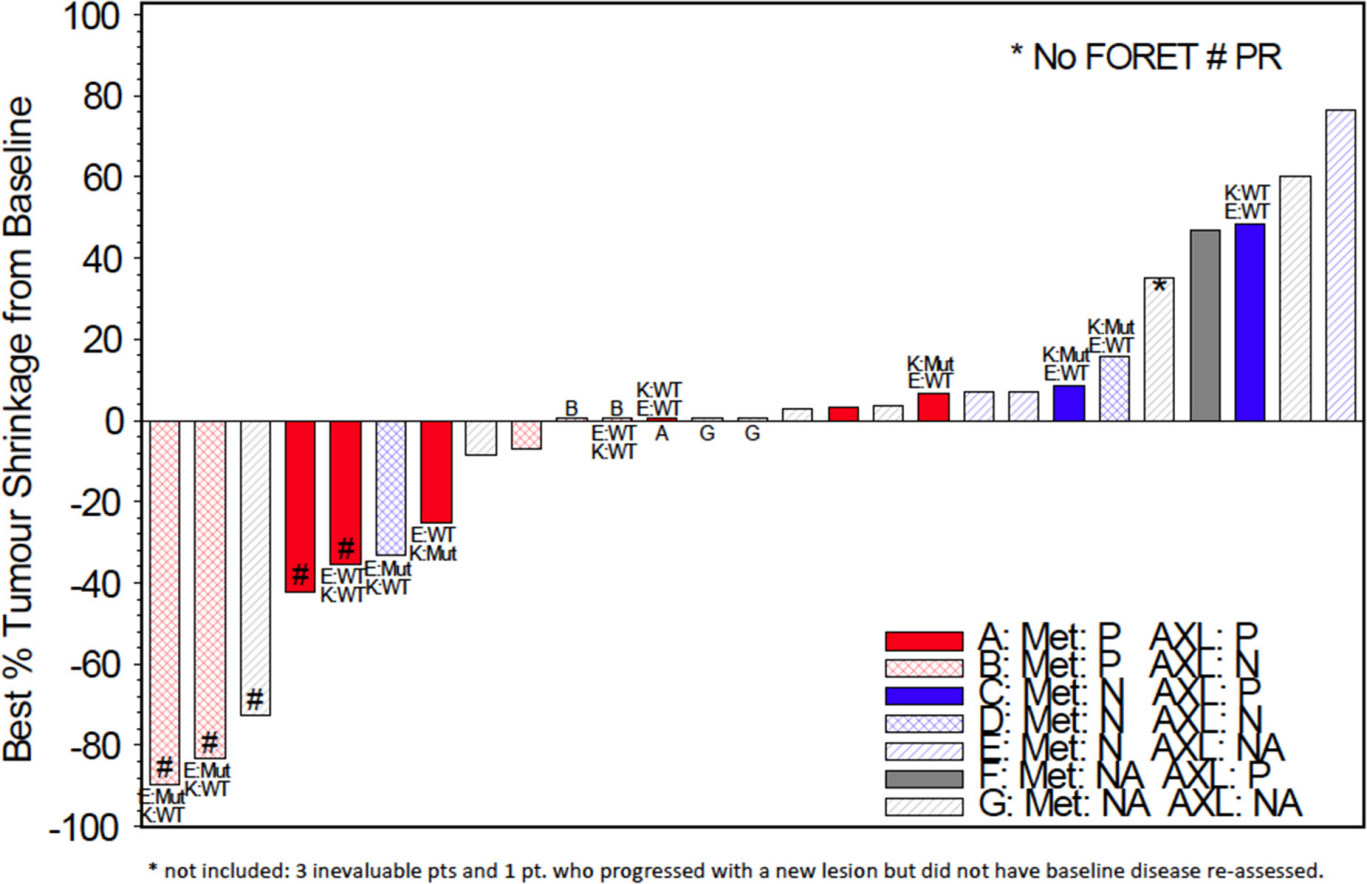
Tumor shrinkage from baseline by genotype and immunohistochemical status (*EGFR*, *KRAS*, MET, AXL) (N=27) K;KRAS; E:EGFR; Mut: Mutant; WT: Wild Type; # PR: partial response; P: positive; N: negative; NA: not assessable.

**Figure 2:**
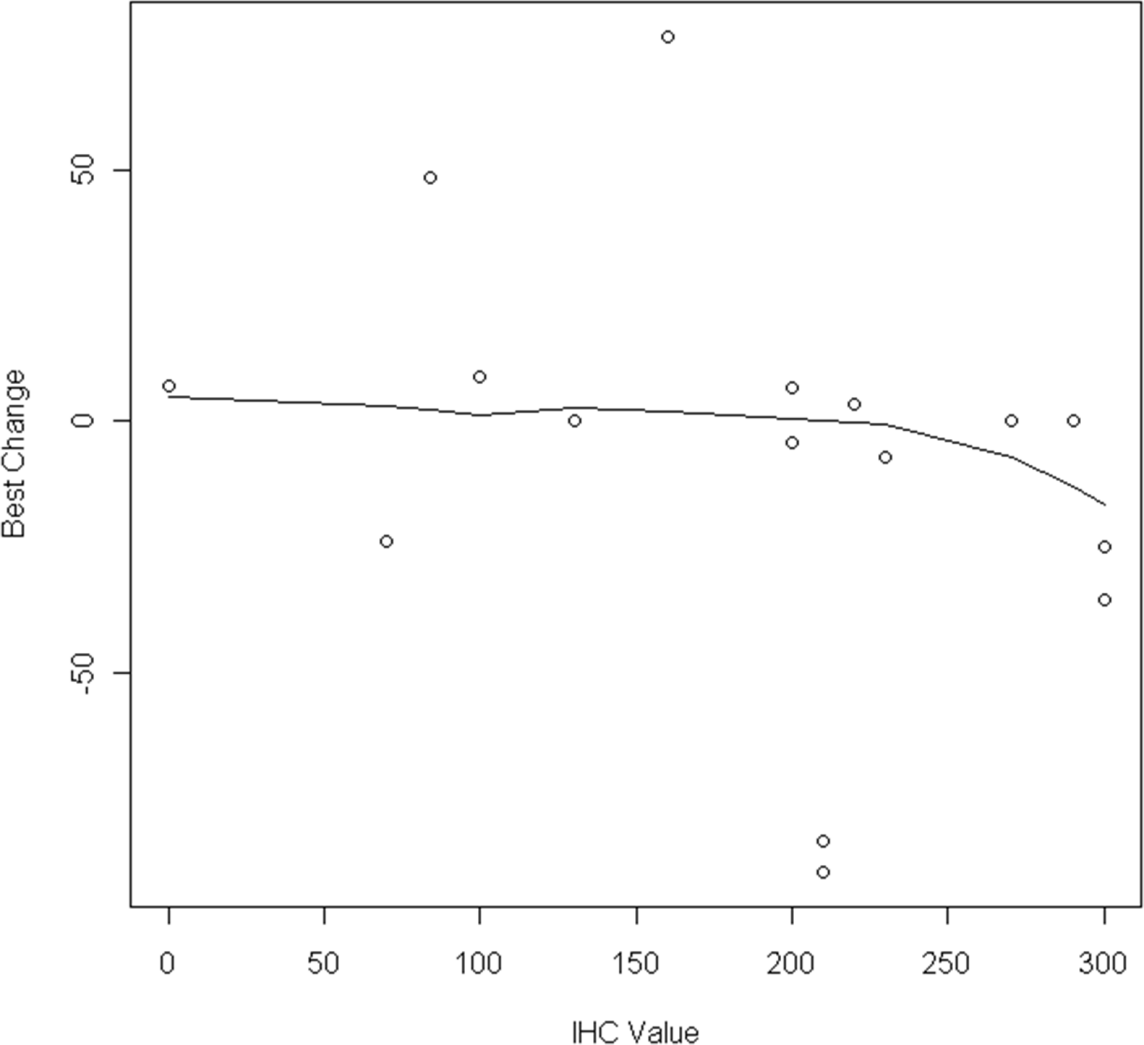
Percent tumor shrinkage by MET H-score (N=19)

The association between *EGFR*, *MET, KRAS* and AXL status and outcomes were examined, shown in Figure 1. Although trial eligibility mandated submission of tissue for genotyping for all patients, only 11/31 patients had sufficient tissue for successful analysis, with 3 *EGFR* mutations and 4 *KRAS* mutations identified. No cases of *MET* mutation, amplification nor *EGFR* amplification were identified. The following results are uncontrolled for *EGFR* genotype (2/5 responses in *EGFR* mutant, 1/5 wild-type, 2 unknown). Exploring MET expression, 11/18 cases were deemed positive, and one third had responses (4/18). While not statistically significant, there appeared to be an association between tumor shrinkage and MET H-score levels above 200 (p=0.12, see Figure 2). Nine of 16 cases were positive for AXL by immunohistochemistry, and 2/9 had response to therapy. Interestingly there was a negative association between AXL staining and *EGFR* mutation (p=0.02); 3 of 4 patients with *KRAS* mutations had positive AXL expression. Indeed, the only AXL-positive cases with evidence of tumor shrinkage also had positive *MET* expression. Lower levels of baseline serum HGF were associated with disease progression as best response (p=0.02).

## DISCUSSION

In this dose escalation study of combination foretinib and erlotinib in advanced pre-treated NSCLC patients, the recommended phase II dose was determined to be foretinib 30 mg daily plus erlotinib 150 mg daily in patients that tolerate a 14 day run-in of full dose erlotinib. Because of incremental and potentially overlapping toxicities, dose reductions and dose holds were common with the combination. There was no confirmed evidence of PK interaction between agents. The response rate in this unselected population was 17.8%, uncontrolled for *EGFR* mutation status. While two responders had documented tumor EGFR mutations, one had wild type disease and the rest insufficient tissue for genotyping. Exploratory biomarker analysis was limited by results available in only 18 patients, but suggests a non-significant association between treatment response and MET protein expression. AXL expression in available baseline samples was inversely associated with the presence of *EGFR* mutations and not associated with response. Lower baseline levels of HGF were associated with disease progression.

The interest and progress in targeting MET in lung cancer is growing. A number of agents have shown activity in patients with MET-activated lung cancers, such as crizotinib, cabozantinib and capmetinib in patients with tumoral exon 14 skipping mutations and/or *MET* amplification [10]. Combination EGFR kinase and MET inhibitor studies have been developed in both *EGFR* mutant and unselected patient populations. Initial trials of tivantinib and onartuzumab with erlotinib in unselected patients or those with high MET protein expression have been disappointing [19, 20]. Development of other novel agents in this setting has also been challenging. Combination dacomitinib and crizotinib in a dose escalation/expansion study of 70 pretreated advanced lung cancer patients yielded substantial toxicity and only 1 response (1%) [21]. No association with *MET, EGFR* nor MET/EGFR protein expression was seen. A similar study with erlotinib and crizotinib in pretreated nonsquamous lung cancer patients demonstrated significant toxicity, increased erlotinib exposure from crizotinib pharmacokinetic interaction and a RP2D of erlotinib 100 mg daily plus crizotinib 150 mg BID, below the single agent RP2D of either drug [22]. Responses were seen in only 2 patients with *EGFR* mutant tumours. A phase I/II study of combination cabozantinib plus erlotinib reported responses in 5/61 patients (8%, genotype not reported) in dose escalation but none in the dose expansion phase [23]. Interestingly, the randomized ECOG-ACRIN 1512 phase II study in pretreated *EGFR* wild type NSCLC patients demonstrated a doubling of PFS with cabozantinib/erlotinib compared to erlotinib alone, (4.7 v 1.8 months, HR 0.37, 95% CI 0.25-0.53, one-sided p=0.0003) [24].

Greater success has been seen in studies of the *EGFR* mutant lung cancer population in the setting of acquired EGFR kinase resistance, and those that select for MET activation (e.g. amplification, high copy number, mutations). Exploration of several combinations are ongoing in this population with evidence of activity, including erlotinib/cabozantinib with restoration of responses in 8% [25], gefitinib/capmatinib with a response rate of 18% in those selected for high MET protein expression or gene copy number (GCN), and up to 30% response in those with MET GCN ≥6 [26], and gefitinib/tepotinib with responses seen in 5/18 patients also selected by MET expression or GCN [27]. Trials are ongoing with other novel combinations including erlotinib/capmatinib [28; NCT01911507], EGF816/capmatinib [NCT02335944], osimertinib/salvolitinib [NCT02143466], erlotinib/emibetuzumab[NCT01900652] and more.

Limitations of this study include its small size, overlapping drug-related toxicities and insufficient tissue samples for routine biomarker assessment in all patients. While some may criticize the study population for being molecularly unselected patients receiving initial EGFR kinase inhibitor therapy, rather than *EGFR* mutant NSCLC patients with acquired kinase inhibitor resistance, we believed the population to be appropriate for this dose escalation study. However, moving forward with further development, consideration must be given to the value of additional MET, AXL and other kinase inhibition with foretinib added to erlotinib in the *EGFR* mutant population with acquired EGFR kinase therapy resistance and those with primary resistance to *EGFR* inhibitors. This may also allow greater flexibility in doses used, for example in our study we targeted early escalation to full dose erlotinib at 150 mg daily given the unselected nature of the patient population studied. Future studies of foretinib and erlotinib should be in molecularly selected patients.

Robust conclusions from our exploratory biomarker studies are limited by small numbers and adequacy of tissue. Despite mandatory submission of tumour samples for all participants, only 11, 16 and 18/31 patients had sufficient tissue for genotyping, AXL and MET expression respectively. Responses were seen in 5 patients, 2 with *EGFR* mutant tumours, 1 with *EGFR* wild type but 2 had insufficient tissue for genotyping. The additional responses could have been explained by missed *EGFR, MET* mutation or *ROS-1* rearrangement, all potential targets of erlotinib or foretinib, but there are insufficient plasma or tissue samples for further testing in this cohort. Even with small numbers, a potential negative association between AXL and *EGFR* mutations was seen in our study prior to EGFR kinase exposure. However, our findings do not reflect the biology of those with *EGFR* mutant lung cancer and acquired EGFR kinase resistance, where these biomarkers may play a more important role [16]. The use of MET as a predictive biomarker has also been challenging, with no clear consensus as to how or even if immunohistochemistry should be used beyond potential screening for genomic alterations [20, 29]. While we did not detect MET gene alterations in our study, the potential association with response and higher MET protein expression further support development of these agents in populations with evidence of MET activation. It must be acknowledged that while 4 of 5 patients with partial response had tumoral MET expression in our study, 2 of these also had *EGFR* sensitizing mutations and were TKI naïve. Emerging studies suggest these populations may be better defined through the presence of *MET* activating mutations and high gene copy number or amplification [10, 26].

While the combination of foretinib and erlotinib is feasible at the recommended phase II dose defined in this study, it is unclear if the potential efficacy in this population can outweigh incremental toxicity based on this small early phase trial. The value of combination targeted therapy in the absence of oncogene addiction or signaling dependence remains challenging. Alternatives such as checkpoint inhibition and other immune-mediated therapies may be preferable, unless clear reliance on multiple signaling pathways can be demonstrated for individual tumors. Future studies will need larger sample size and biomarker stratification to allow exploration of efficacy in *EGFR* mutant versus wild type NSCLC, and also in the setting of primary and acquired resistance to EGFR inhibitors. In addition, greater understanding of the functional activation of MET and impact of genomic aberrations will be important in understanding why selected MET inhibitors fail or succeed in lung cancer and other tumor types reliant on MET-mediated signaling.

## MATERIALS AND METHODS

This Canadian Cancer Trials Group study was conducted at 4 participating Canadian cancer centers, the Princess Margaret Cancer Centre (Toronto, Ontario), the British Columbia Cancer Agency (Vancouver, British Columbia), the Juravinski Cancer Centre (Hamilton, Ontario) and the Ottawa Hospital Cancer Centre (Ottawa, Ontario). All sites received institutional review board approval for study conduct. Written informed consent was obtained from all individual study participants.

### Patient population

Patients with advanced non-small cell lung cancer were eligible to participate in the trial if they had: (1) histologically or cytologically confirmed NSCLC; (2) one prior regimen of chemotherapy for advanced disease that had failed and were eligible to receive erlotinib; (3) positive or unknown tumor EGFR protein expression (per product label); (4) archival tissue available for analysis (including repeat sampling prior to registration); (5) had measurable disease by RECIST 1.1; (6) had an Eastern Cooperative Group performance status (ECOG PS) of ≤ 2; (7) had adequate renal (serum creatinine < 1.5 times the upper institutional limit) and hepatic (serum alanine aminotransferase or aspartate aminotransferase < 2 times the upper institutional limit) function; and (8) the ability to provide written informed consent. Patients were excluded if they had: (1) untreated or uncontrolled cardiovascular conditions including resting systolic blood pressure >150 mm Hg and/or diastolic pressure > 100 mm Hg; (2) more than two prior chemotherapy regimens for metastatic disease; (3) prior treatment with anti-EGFR agents; (4) symptomatic or untreated brain metastasis. In December 2010, the protocol was amended to exclude patients with pre-existing thromboembolic disease, based on data from other studies suggesting a potential increase in risk with foretinib.

### Study design

In this dose-finding phase I trial, escalating doses of foretinib were added to standard dose erlotinib as continuous daily dosing. Patients were permitted to remain on study treatment until the development of disease progression, unacceptable toxicity or withdrawal of consent. To facilitate pharmacokinetic studies and ensure tolerability of standard therapy, patients received erlotinib alone daily for 14 days during cycle 1, and foretinib was initiated on day 15, cycle 1 if erlotinib was well tolerated. Daily dosing for both agents thereafter was continuous. At the starting dose level, the first patient entered was observed for 28 days prior to enrolling other patients at that dose level.

### Dose escalation

Toxicity was evaluated continuously according to the Common Terminology Criteria for Adverse Events (CTCAE), Version 4.0. If no dose limiting toxicity (DLT) was seen in the first 3 patients at a given dose level, escalation to the next dose level occurred after review by the NCIC CTG study physicians and investigators (see Table 4). If 1/3 experienced DLT, at least 3 additional patients were treated for a total of 6 evaluable patients treated at that dose level. If 2/3 or 2/6 patients experienced DLT, dose escalation was to be stopped and that dose declared the maximum tolerated dose (MTD), with the next lower dose to be declared the recommended phase II dose for foretinib in combination with erlotinib. While designed as a 3+3 dose escalation study, 3+4 was allowed to ensure that there would be a minimum of 3 evaluable patients without having to reopen a cohort to replace a patient. Intra-patient dose escalation was not allowed. Patients that were not evaluable for DLTs in cycle 1 were replaced to ensure an adequate number of patients evaluable for toxicity. DLT was defined as grade 3 or worse non-hematologic toxicity (excluding alopecia, inadequately managed diarrhea, nausea, vomiting, rash or hypertension), treatment delay of more than 14 days for cycle 2, and toxicities of concern to investigators or the NCIC CTG that occurred within cycle 1 and were considered possibly, probably or definitely related to either or both study drugs.

**Table 4:** Summary by dose level (N=31)

Dose Level (foretinib/erlotinib in mg)	N	Inevaluable for DLT	DLT	Action taken
DL1 (30/100)	4	1	None	Open DL1
DL2 (30/150)	8	1	1 gr 3 rash with dose delay, reduction	DL2 expanded, then open DL3
DL3 (45/150)	4	0	None	DL3 expanded for suspected PK interaction
DL3 expansion cohort	3	0	None	DL2, DL1 expanded for unconfirmed PK interaction
DL2 expansion cohort (30/150)	7	3	1 gr 3 mucositis, neck pain (delay, dose reduction, admission) 1 gr 3 fatigue, nausea (delay)	DL2 declared RP2D
DL1 expansion cohort (30/100)	5	1	None	

DL: dose level; gr: grade; PK: pharmacokinetic; RP2D: recommended phase 2 dose.

### Evaluation on treatment

Patients underwent history and physical examination, performance status, measurement of vital signs, routine hematology and biochemistry studies, thyroid studies and tumor imaging with computed tomography scans at baseline. Blood pressure monitoring and bloodwork was repeated weekly during cycle 1 and then on day 1 of each subsequent cycle. Patients were assessed on day 1 of each subsequent cycle and as needed for toxicity management. Tumor assessment with imaging was performed at every second cycle (8 weeks). Urinalysis was performed on day 1 of each cycle, ophthalmologic examination every 12 weeks, and pharmacokinetic sampling on day 14 and 28 of cycle 1.

### Pharmacokinetic analyses

The maximum observed concentration (C_max_), time to reach C_max_ (T_max_), the area under the plasma concentration time curve over 24 hours of dosing (AUC0-24), and oral clearance (CL/F) for both foretinib and erlotinib were estimated by non-compartmental pharmacokinetic methods, from observed concentration-time profiles and log-linear trapezoidal algorithm (AUC).

Patients were required not to smoke or chew tobacco products for at least 14 days prior to study entry, and to abstain at least until the completion of pharmacokinetic sampling on day 28 of cycle 1.

### Pharmacodynamic analyses

Participants were required to submit archival tumor samples. Tumor genotyping was performed using Sequenom MassARRAY, using the OncoCarta Panel v1.0 (San Diego CA), with verification using Sanger sequencing. Immunohistochemistry was performed for AXL and MET, using the human AXL affinity purified polyclonal goat IgG antibody (R&D systems, AF154, Minneapolis MN), and MET was stained with the anti-total MET (SP-44) rabbit monoclonal antibody (Ventana Medical Systems, Tucson AZ) using the Benchmark XT autostainer. Staining intensity (0-3+) and percent of cells stained were used to calculate the H-score, [1 × (% cells 1+) + 2 × (% cells 2+) + 3 × (% cells 3+)]. H-scores ≥100 were defined as positive for AXL, and ≥ 200 positive for MET (median H-score). Circulating baseline and on-treatment levels of HGF were measured through ELISA (R&D Systems, Minneapolis, MN).

### Statistical analysis

The primary endpoint was to determine the recommended phase II dose of daily oral foretinib in combination with standard erlotinib therapy. Descriptive statistics of safety, DLT, response, duration of response, pharmacokinetic measures and pharmacodynamic data were summarized. Correlation between toxicity and outcomes with pharmacokinetic and pharmacodynamic measures was explored through waterfall plot and Fisher’s exact test.
